# Extensive gene loss in the plastome of holoparasitic plant *Cistanche tubulosa* (Orobanchaceae)

**DOI:** 10.1080/23802359.2020.1787273

**Published:** 2020-07-08

**Authors:** Wanqi Xu, Haimei Chen, Lixia Tian, Mei Jiang, Qiaoqiao Yang, Liqiang Wang, Bashir Ahmad, LinFang Huang

**Affiliations:** a Key Research Laboratory of Traditional Chinese Medicine Resources Protection from Ministry of Education, Engineering Research Center of Chinese Medicine Resource, National Administration of Traditional Chinese Medicine, Institute of Medicinal Plant Development, Chinese Academy of Medical Sciences, Peking Union Medical College, Beijing, China; b Center for Biotechnology and Microbiology, University of Peshawar, Peshawar, Pakistan

**Keywords:** *Cistanche tubulosa*, plastome, gene loss, holoparasitic plant, phylogenetic analysis

## Abstract

Extensive photosynthetic gene loss and rapid evolutionary rate occur in the plastomes of parasitic plants. The holoparasitic plant *Cistanche tubulosa* of Orobanchaceae is an important medicinal resources that are distributed in arid areas. In this study, the complete plastome of *C. tubulosa* has been sequenced, assembled and analyzed. The total plastome of *C. tubulosa* was 75,375 bp in length, consisting of a pair of inverted repeats (IRs, 6,593 bp), a large single-copy region (LSC, 32,470 bp) and a small single-copy region (SSC, 29,719 bp). It contained 24 intact protein coding genes, nine pseudogenes, and 44 missing genes. In addition, all the protein-coding genes, which were related to photosynthesis and energy production, were pseudogenised or lost. Four rRNA genes and 24 tRNA genes were intact meanwhile five tRNA genes were missing. Phylogenetic tree indicated that *C. tubulosa* was closely related to *C. phelypaea*. Our results may improve understanding of the plastome organization, classification, and evolution of parasitic plants.

Orobanchaceae is a special family that comprises all level growth behavior plants consisting of nonparasitic, hemiparasitic and holoparasitic plants (Xi et al. 2013). *Cistanche tubulosa*, a holoparasitic plant of the Orobanchaceae, absorbs water and organic and inorganic nutrition from the roots of its hosts. *Cistanche tubulosa* is traditionally used as nourishing herbs. A lot of compounds have been isolated from *C. tubulosa*, including phenylethanoid glycosides, carbohydrates, lignans, iridoids, echinacoside, verbascoside, chlorogenic acid, acteoside, and luteoloside (Yong and Peng-Fei 2009; Fu et al. 2018; Gao et al. 2019). These isolated compounds have exhibited abundant pharmacologic effects, such as neuroprotective, immunomodulatory, anti-senescence, anti-inflammatory, anti-osteoporosis, hepatoprotection, anti-oxidative, anti-bacterial, anti-tumour and glucose-tolerance-improving effects (Morikawa et al. 2019). Compared with the more than 3000 plastomes of autotrophic organisms that can be obtained from the National Center for Biotechnology Information database, data from the plastomes of parasitic plants are limited. To depict the characteristic of gene losses and provide new insights into the overall evolutionary process, we analyzed the plastome of *C. tubulosa*.

Fresh scale leaves of *C. tubulosa* were collected from the Hotan Prefecture (Xinjiang Uygur Autonomous Region), China (78°12′36″E, 36°19′12″N). The voucher specimens were deposited in the herbarium of Institute of Medicinal Plant Development, Chinese Academy of Medical Sciences, Peking Union Medical College (IMD), with accession numbers of XJ20170502. Approximately 500 ng DNA was used to construct a library with an insert size of 400 bp and sequenced according to the manufacturer’s instructions for HiSeq 4000 platform. The clean paired-end reads were filtered against all the plastomes of plants recorded in the GenBank database by using BLASTn with an e-value cutoff of 1e − 5. The extracted reads were assembled using SPAdes (v. 3.10.1) (Bankevich et al. 2012). The boundary region was validated using primer pairs spanning the boundary region followed by PCR amplification of the regions and Sanger sequencing of the PCR production. Genes were annotated by using CpGAVAS2 web service (Shi et al. 2019) and edited manually by using Apollo genome editor (Lewis et al. 2002). The circle map of plastome was generated using OrganellarGenomeDRAW (Lohse et al. 2013), and GC content was analyzed using CGView Server (Grant and Stothard 2008). In comparison with the nonparasitic plant of Orobanchaceae *Rehmannia glutinosa* (NC_034308), genes that were similar to known protein-coding genes but truncated or contained one or more frameshift mutations, were classified as pseudogenes (Cusimano and Wicke 2016; Xi et al. 2013). The genome assembly and annotation results were deposited in GenBank, with accession numbers of MN614130.

The plastome of *C. tubulosa* was 75,375 bp in length and showed a typical quadripartite structure, including a pair of IRs (6593 bp) separated by LSC (32,470 bp) and SSC (29,719 bp) region. The total GC content was 34.95% and the SSC region had a higher GC content (38.00%) than the IR (33.22%) and LSC (32.86%) region. The plastome of *C. tubulosa* retained 27 intact protein-coding genes, nine genes became pseudogenes, and 44 genes were missing completely. All genes related to encoding photosynthetic proteins were pseudogenised or lost, which supports the holoparasitic lifestyle of *C. tubulosa*. All four rRNAs were universally localized in the SSC region. A total of 24 tRNAs remained, and up to five tRNAs were lost throughout the evolution.

To analyze the phylogenetic position of *C. tubulosa* within the Orobanchaceae famliy, a molecular evolutionary tree was constructed using 36 species. Twelve shared protein sequences were concatenated and aligned using the ClustalW program (Thompson et al. 2002). The phylogenetic tree was constructed using the Randomized Axelerated Maximum Likelihood (RAxML) software (Stamatakis 2014) and the ML method, with *Arabidopsis thaliana* and *Nicotiana tabacum* as the outgroups. The phylogenetic tree showed that *C. tubulosa* and *C. phelypaea* were grouped together (Figure 1).

**Figure 1.**
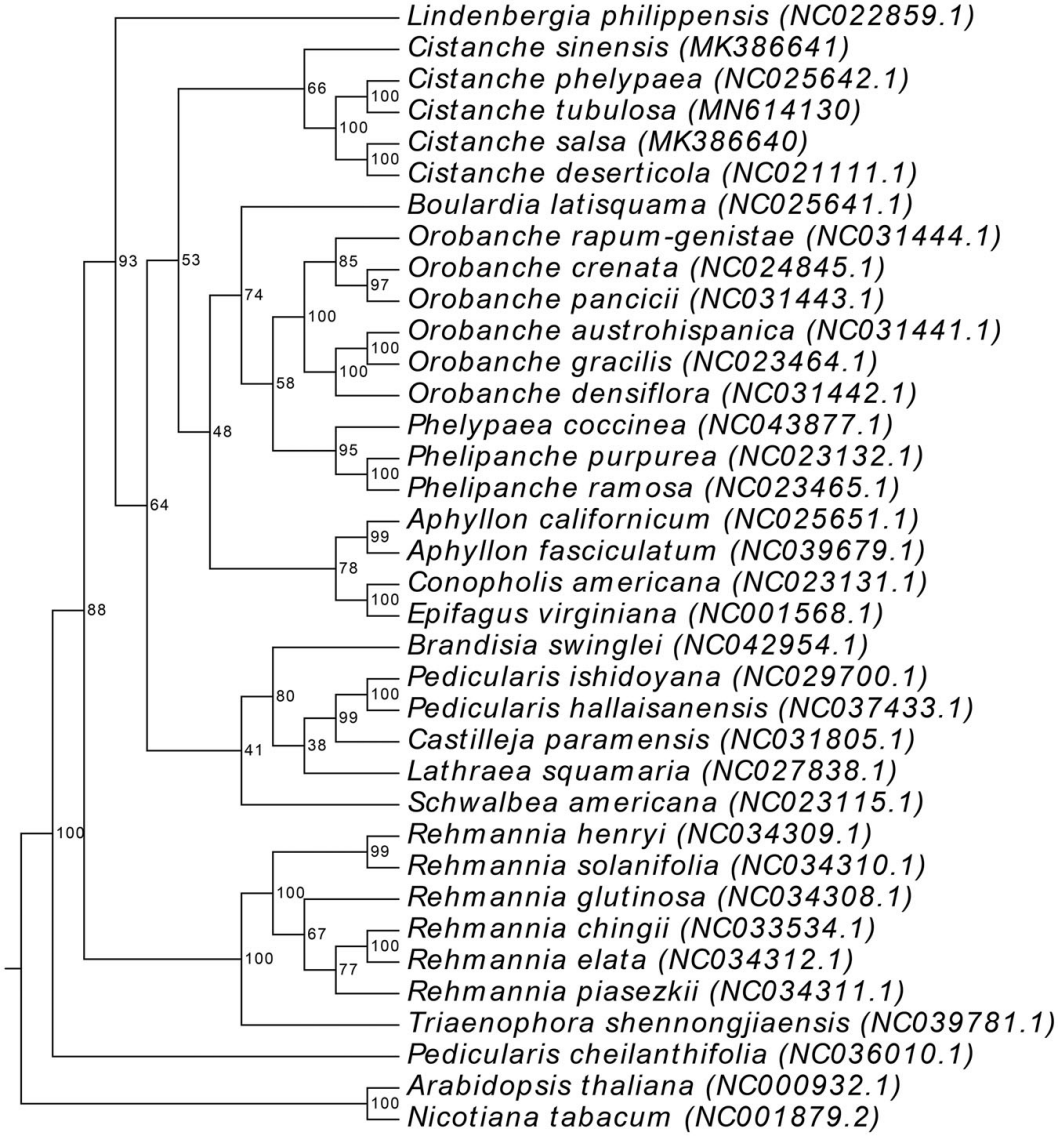
Phylogenetic tree based on 36 complete plastomes of Orobanchaceae.

## Data Availability

The data that support the findings of this study are deposited in GenBank, with accession numbers of MN614130. https://www.ncbi.nlm.nih.gov/nuccore/MN614130.1/.
